# miR-378a-5p inhibits the proliferation of colorectal cancer cells by downregulating CDK1

**DOI:** 10.1186/s12957-021-02166-w

**Published:** 2021-02-19

**Authors:** Kai Li, Jieling Zhang, Mingkang Zhang, Yaohua Wu, Xinyu Lu, Yiping Zhu

**Affiliations:** 1 Department of Oncology, The First Affiliated Hospital of Wannan Medical College, Wuhu, 241002 China; 2 Department of Clinical medicine, Wannan Medical College, Wuhu, 241002 China; 3 Anhui Province Key Laboratory of Biological Macro-molecules Research, Wannan Medical College, Wuhu, 241002 China

**Keywords:** miR-378a-5p, CDK1, CRC, Proliferation, Apoptosis

## Abstract

**Background:**

MicroRNAs (miRNAs) play an important role in tumor occurrence. The role of miR-378a-5p and CDK1 in colorectal cancer (CRC) was investigated in this study.

**Methods:**

Investigation of TCGA database and the detection of miR-378a-5p expression in colorectal cancer pathological tissues and colorectal cancer cell lines were undertaken by using qRT-PCR. We performed cell function experiments (CCK-8 assay, EdU assay, colony formation assay, wound healing assay, transwell assay, cell apoptosis assessment, and cell cycle assessment) and nude mouse tumor formation experiments to evaluate the effects of miR-378a-5p on proliferation, metastasis, and invasion to explore the role of miR-378a-5p in vivo and in vitro. Next, through TCGA database, immunohistochemical staining of pathological tissues, and cell function experiments, the role of the target gene CDK1 of miR-378a-5p was verified by database prediction, and dual luciferase reporter gene experiments in colorectal cancer cells were performed. Finally, whether upregulation of CDK1 restores the inhibitory effect of overexpression of miR-378a-5p on the proliferation of CRC cells was studied by overexpression of CDK1.

**Results:**

Bioinformatic analysis showed significant downregulation of miR-378a-5p levels in colorectal cancer (CRC). Cell function experiments and tumor xenograft mouse models confirmed the low expression of miR-378a-5p within CRC tissues, which indicated the tumor suppressive role of miR-378a-5p in CRC. To better explore the regulation of miR-378a-5p in CRC, we predicted and validated cell cycle-dependent protein kinase 1 (CDK1) as the miR-378a-5p target gene and observed that miR-378a-5p suppressed CRC cell proliferation by targeting CDK1.

**Conclusion:**

The results of this study help to elucidate the mechanism by which miR-378a-5p can be used as a tumor marker to inhibit the growth of colorectal cancer and CDK1, which is related to the prognosis of colorectal cancer patients. MiR-378a-5p inhibits CRC cell proliferation by suppressing CDK1 expression, which may become a possible therapeutic target for treatment of CRC.

## Introduction

Colorectal cancer (CRC) is a frequently occurring malignant tumor occurring in the digestive system. According to the *Global Cancer Statistics 2018*, globally there were 1.8 million newly diagnosed CRC cases and 881,000 CRC deaths in 2018, accounting for approximately 1/10 of all cancer cases and deaths. Overall, CRC ranked second as a cause of cancer death [1, 2]. Despite the increasing number of new therapies for CRC, the treatment outcome for patients with advanced CRC is not satisfactory. Therefore, it is of great significance to develop new treatments.

Tumor cells are mainly characterized by uncontrolled growth, disturbed cell cycle, and indefinite proliferation ability, in which disturbance of the cell cycle may lead to tumorigenesis. Cell cycle-dependent protein kinases and cyclins precisely regulate the cell cycle, while changes in their concentrations can affect the cell cycle, thereby promoting cell proliferation or leading to apoptosis [3–5]. Cell cycle-dependent protein kinase 1 (CDK1), which belongs to the serine/threonine-protein kinase family, is mainly active in the late G2 and early M phases, and high expression of active CDK1 can promote the G2/M transition and accelerate the growth of tumor cells [6–8]. CDK1 plays an oncogenic role in various tumors, such as liver cancer, melanoma, bladder cancer, and breast cancer [9–12], indicating its potential as a therapeutic target for treating different cancers. However, the regulatory mechanism of CDK1 in CRC has not been fully studied and needs further exploration.

MicroRNAs (miRNAs), noncoding small RNAs with high evolutionary conservation (with a length of approximately 19–23 nucleotides), modulate posttranscriptional gene expression and play a vital role in regulating human cancer and affect tumor cell proliferation, apoptosis, and migration [13].

Recently, miR-378a-5p was shown to suppress renal cell carcinoma (RCC) development [14], which is associated with patient prognosis; in addition, it has been reported to have an antiapoptotic function and regulate smooth muscle cell migration and invasion in breast cancer [15]. Of note, miR-378a-5p has been reported to be highly correlated with CRC prognosis [16]; however, there are few reports on the mechanism of action of miR-378a-5p in CRC, which requires further study.

To explore the role of miR-378a-5p in colorectal cancer and discover new treatments for colorectal cancer, we conducted this study. The present work discovered that miR-378a-5p showed low expression levels in CRC, which suppressed CRC cell proliferation and apoptosis resistance. Furthermore, miR-378a-5p downregulated CDK1 levels to suppress CRC development.

## Materials and methods

### Tissue sample collection

The present work gained approval from the Medical Ethics Committee of Yijishan Hospital of Wannan Medical College (Wuhu, China). Each case provided the informed consent for participation. A total of 108 paraffin sections were collected from the Pathology Department, Yijishan Hospital Affiliated to Wannan Medical College, and 22 CRC tissue samples with matched adjacent non-carcinoma tissue samples (around 5 cm apart from cancer edge) were acquired and frozen within liquid nitrogen at once.

### Cell culture and transfection

Various types of CRC cells (SW480, HCT116, SW620, HT-29) were obtained from Cell Bank of Chinese Academy of Sciences (Shanghai, China); meanwhile, the colon epithelial cells (NCM460) were obtained from the Affiliated Comprehensive Cancer Center of Drum-Tower Hospital of Medical School of Nanjing University. The CRC cells were cultured in RPMI-1640 medium (Gibco, Carlsbad, CA, USA) containing 10% fetal bovine serum (Gibco, Carlsbad, CA, USA) within the incubator under 5% CO_2_ and 37 °C conditions at the Roswell Park Memorial Institute. Then, the Lipofectamine 3000 Kit (Invitrogen, Carlsbad, CA, USA) was used to transfect cells in accordance with manufacturer instructions. The small interfering RNA (siRNA) CDK1, together with the corresponding negative control (NC), was prepared by Guangzhou Ribo Biotechnology Co. Ltd (Guangzhou, China), and its sequences are listed below: siRNA1: GGAACTTCGTCATCCAAAT, siRNA2: GTACTGCAATTCGGGAAAT, siRNA3: GGTTATATCTCATCTTTGA. In addition, the CDK1 overexpression plasmid as well as the empty plasmid was provided by GenePharma Co. Ltd (Shanghai, China), whereas the miR-378a-5p inhibitor and miR-378a-5p mimic, together with corresponding NCs were provided by Guangzhou Ribo Biotechnology Co. Ltd. (Guangzhou, China).

### Reverse transcription-quantitative polymerase chain reaction (RT-qPCR)

TRIzol reagent (Invitrogen, CA, USA) was used to extract the total tissue and cellular RNA in accordance with specific protocols. Total RNA was reverse transcribed to complementary DNA following the protocols of the RT Kit (Takara, Dalian, China). The RT reaction program used was 42 °C for 60 min and 70 °C for 10 min. Real-time fluorescence PCR was carried out with a qPCR Kit (Takara, Dalian, China). U6 and GAPDH were used as internal references for miR-378a-5p and CDK1, respectively. The primer sequences of miR-378a-5p and U6 were designed by Ruibo: GAPDH, forward: GAACGGGAAGCTCACTGG, reverse: GCCTGCTTCACCACCTTCT; CDK1, forward: GGTTCCTAGTACTGCAATTTTCG, reverse: TTTGCCAG.

The results were calculated by the 2^−ΔΔCt^ method.

### Cell counting kit-8 (CCK-8) assay

Cell viability was assayed using Cell Proliferation Kit (Keygen Biotechnology Co. Ltd. (Nanjing, China)). CRC cells were detached and inoculated into the 96-well plates at 2 × 10^4^ cells/well, and the adhering cells after 12-h cell culture were transfected. At 12, 24, 36, 48, and 60 h of transfection, every group was added with 10 mL reagent to detect cell proliferation; then, cells were incubated for another 2 h, and the absorbance (OD) was detected at 450 nm.

### 5-Ethynyl-2’-deoxyuridine (EdU) assay

CRC cells (1 × 10^5^/ well) at logarithmic stage were seeded into the 96-well plates to culture until the normal growth stage. Thereafter, cells were labeled with EdU and stained with Apollo and DNA as per the manufacturer’s instructions (Guangzhou Ribo Biotechnology Co. Ltd., Guangzhou, China); at last, the confocal laser scanning microscope was utilized to observe cells.

### Colony formation assay

CRC cells (200 cells/well) after transfection were grown into the 6-well plates and further incubated for 2 weeks under 37 °C. At last, 4% paraformaldehyde was used to fix cells, while 0.1% (w/v) crystal violet was used to stain cells. The Image-Pro Plus 5.0 software was employed for counting cell colonies.

### Wound-healing assay

The single straight wound was made using a sterilized 10-μL pipette tip at the bottom of 6-well plates full of transfected CRC cells with cell debris removed by washes with phosphate buffer saline (PBS). Then, every well was added with RPMI-1640 containing 2% FBS, followed by further cell culture under 5% CO_2_ and 37 °C conditions. The width of the scratch was measured with the ImageJ software and an inverted optical microscope. The scratch width measured initially was assigned to be the original scratch width, and the scratch width measured again after 48 h was used as final scratch width. Relative scratch width was calculated by the ratio of final scratch width to the original one.

### Transwell assay

At 24 h after transfection of CRC cells, 100 μL of the cells diluted to 1 × 10^5^ with serum-free RPMI-1640 medium was plated at apical side of a transwell chamber, whereas 600 μL of 20% RPMI-1640 medium was put at basolateral side. After 24 h of cell incubation under 37 °C and 5% CO_2_ conditions, the 4% paraformaldehyde was used to fix the chamber, while 0.1% crystal violet was used for staining. Thereafter, a cotton swab was used to remove upper chamber cells. Finally, migrating cells were photographed and counted with an inverted optical microscope.

### Immunohistochemical (IHC) staining

CDK1 expression was determined with the CDK1 antibody (1:100, Abcam, USA). Xylene and alcohol were used for dewaxing, 3% H_2_O_2_ was used to remove endogenous catalase, and citrate buffer was added; the sample was placed in a microwave oven and cooked to expose the antigen site, after which serum blocking was performed. After incubation, the antibody and the secondary antibody were used to develop the color, and the samples were mounted. Each tissue section was rated using a light microscope based on the staining level (0, 1, 2, and 3 indicated negative, yellowish, light brown, and dark brown staining, respectively) and the extent of positivity (1, 2, 3, and 4 represented 0–25%, 26–50%, 51–75%, and 76–100%, respectively). Finally, the scores were added for comparison. All IHC sections were evaluated independently by two expert pathologists.

### Dual-luciferase reporter gene assay

Luciferase reporter gene assay was performed to assess the direct binding of miR-378a-5p with CDK1. The CDK1 3′-untranslated region (UTR) that may bind to miR-378a-5p was mutated from GTCAGGAA to CAGTCCTT, and a mutant fragment and an unmutated fragment of the CDK1 3′-UTR were transfected to pmirGLO reporter plasmid, respectively. 293T cells were then inoculated in the 24-well plates, followed by co-transfection with equivalent unmutated or mutated pmirGLO and miR-378a-5p mimic or NC-mimic. Renilla luciferase was adopted to be endogenous control. After 24 h of cell culture, the Dual-luciferase Reporter Gene Assay Kit (Shanghai Beyotime Biotechnology Co. Ltd., Shanghai, China) was applied in detection.

### Cell apoptosis detection

Flow Cytometric Kit (BD Biosciences, CA, USA) was utilized to measure cell apoptosis. In brief, cells after transfection were inoculated into the 12-well plates to further culture for another 12 h. Thereafter, cells were cultivated with serum-free medium for another 24 h for apoptosis induction; later, 0.25% trypsin was used to detach CRC cells, followed by 5–10 min centrifugation at 2000 rpm under ambient temperature. Thereafter, cells were harvested to suspend in the pre-chilled 1 × PBS (4 °C), followed by 5–10 min of centrifugation at 2000 rpm. Then, cells were washed and resuspended into 300 μL 1 × binding buffer, sufficiently blended using 5 μL Annexin-V-fluorescein isothiocyanate (FITC), followed by 15 min of incubation in dark under room temperature. Afterward, the cells were subjected to 5 min of staining using 5 μL propidium iodide (PI) solution before placing on a flow cytometer, and 200 μL 1 × binding buffer added. Finally, flow cytometry (BD Biosciences, CA, USA) was adopted to detect cell apoptosis.

### Cell cycle detection

The cell cycle was determined using Flow Cytometry kit (Keygen Biotechnology Co., Ltd., Nanjing, China) according to specific protocols. In brief, after 24 h transfection, cells were rinsed by PBS, followed by 5 min of centrifugation at 2000 rpm. Then, cells were collected, and the cell density was regulated at 1 × 10^6^/mL. Afterwards, 1 mL single-cell suspension was collected for centrifugation, and the supernatant was fixed with 500 μL of the 70% pre-chilled ethanol overnight at 4 °C. After PBS washes, 500 μL of PI/RNaseA staining solution was added to further incubate in dark for 1 h, and cell cycle was directly examined by flow cytometry (BD Biosciences, CA, USA).

### Tumor xenograft mouse models

Four-week-old male nude mice reared in an SPF animal room (Spf Laboratory Animal Room of Wannan Medical College) were injected with 2*10^6 HT-29 cells in the left armpit. After observing the size of the tumor every day for 15 days, the nude mice were killed by cervical dislocation, and then, the tumors were removed and recorded.

### Western blotting analysis

Radioimmunoprecipitation (RIPA) lysis buffer (Thermo Fisher Scientific, MA, USA) was utilized to extract total cellular protein from CRC cells transfected for 48 h. A bicinchoninic acid (BCA) protein detection kit (Thermo Fisher Scientific, MA, USA) was used to measure the protein content. SDS-PAGE (10%) (Bio-Rad Laboratories, Hercules, CA, USA) was used to separate proteins, after which the proteins were transferred to PVDF membranes. After blocking, the membranes were incubated with primary antibody (1:1000) overnight at 4 °C, followed by incubation with the secondary antibody (1:5000) at room temperature for 2 h before exposure analysis. CDK1 (Abcam, USA) was assessed later, with β-actin as the internal reference (Cell Signalling Technologies, Beverly, MA, USA).

### Statistical methods

Prism (GraphPad Prism 8) was utilized for statistical analysis. The results were expressed as mean ± SD and examined by *t*-test. A difference of *p* < 0.05 was deemed to be statistically significant, *p < 0.05, **p < 0.01, and ***p < 0.001.

## Results

### miR-378a-5p is expressed at lower levels in CRC

**Fig. 1:**
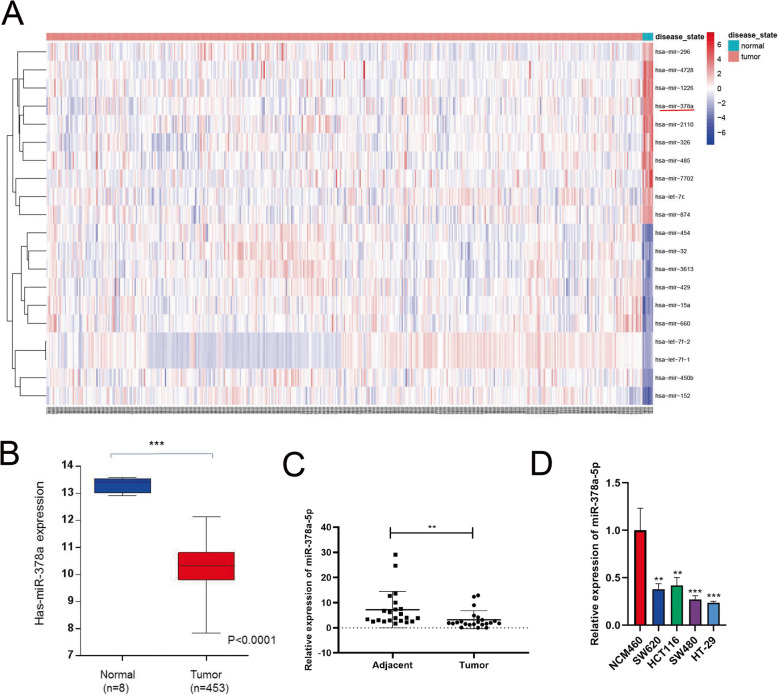
miR-378a-5p is underexpressed in CRC and inhibits colorectal cancer (CRC) tumorigenesis in vivo. **a** Heat map of differentially expressed microRNAs in CRC after statistical analysis of data downloaded from TCGA database. **b** miR-378a-5p levels in CRC tissue samples detected based on TCGA database. **c** miR-378a-5p levels in 22 CRC tissue samples and in matched adjacent noncarcinoma tissue samples assessed by qPCR. **d** miR-378a-5p expression in NCM460, SW620, HCT116, HT-29, and SW480 cells assessed by qPCR. qRT-PCR, real-time quantitative polymerase chain reaction. *p < 0.05, **p < 0.01, ***p < 0.001, and ****p < 0.0001

The Cancer Genome Atlas (TCGA) database was used to analyze microRNAs differentially expressed in CRC, and miR-6833, miR-1248, miR-1277, and remarkable upregulation of miR-378a-5p in CRC tissues were observed (Fig. 1a). We focused on studying the differential expression of miR-378a-5p (Fig. 1b), and the miR-378a-5p levels within the 22 CRC tissue samples together with matched adjacent noncarcinoma tissue samples were assessed through qPCR, which indicated a decreased miR-378a-5p expression within the CRC tissue samples (Fig. 1c). In addition, miR-378a-5p levels decreased in all four CRC cell lines relative to NCM460 cells (Fig. 1d). These findings suggested decreased miR-378a-5p levels in CRC.

### miR-378a-5p impedes the proliferative and migration potentials of CRC

**Fig. 2:**
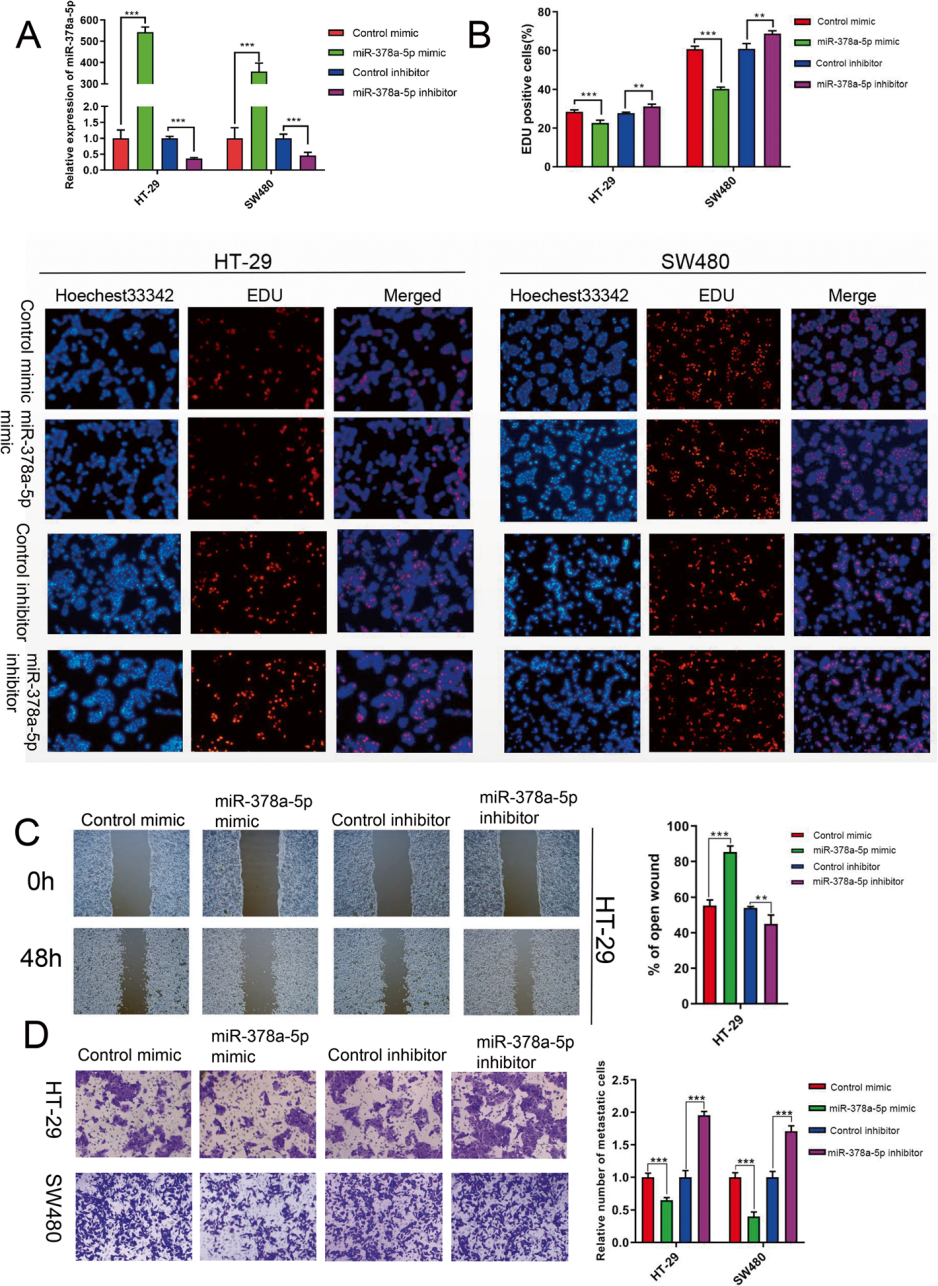
miR-378a-5p suppresses malignant phenotypes of CRC cells. **a** Transfection efficiency was determined using qPCR. **b** The effect of miR-378a-5p on CRC cell proliferation was assessed by an EdU assay. **c** Relative scratch width was measured at 48 h after scratching. **d** Transwell assays were conducted to examine how miR-378a-5p affected cell migration. **p **<** 0.01; *** *p* **<** 0.001

To investigate the impact of miR-378a-5p downregulation on CRC, miR-378a-5p expression was overexpressed and knocked down in the HT-29 and SW480 cell lines, respectively (Fig. 2a), and then, the effect of miR-378a-5p gain/loss-of-function on CRC cell proliferation was examined using an EdU assay. Based on our findings, the overexpression of miR-378a-5p suppressed CRC cell growth and reduced the number of EdU-positive cells, whereas miR-378a-5p knockdown facilitated CRC cell growth (Fig. 2b). We then performed a wound-healing assay after miR-378a-5p overexpression/silencing in HT-29 cells and found that the relative scratch width after overexpression of miR-378a-5p for 48 h was much greater than that after downregulation of miR-378a-5p for 48 h (Fig. 2c). According to transwell assay analysis, the number of migrating HT-29 and SW480 cells decreased when miR-378a-5p was overexpressed, whereas miR-378a-5p suppression increased HT-29 and SW480 cell migration (Fig. 2d). According to these findings, miR-378a-5p suppressed cell proliferation and migration.

### miR-378a-5p promotes colorectal cell apoptosis and inhibits tumor growth in tumor xenograft mouse models

**Fig. 3:**
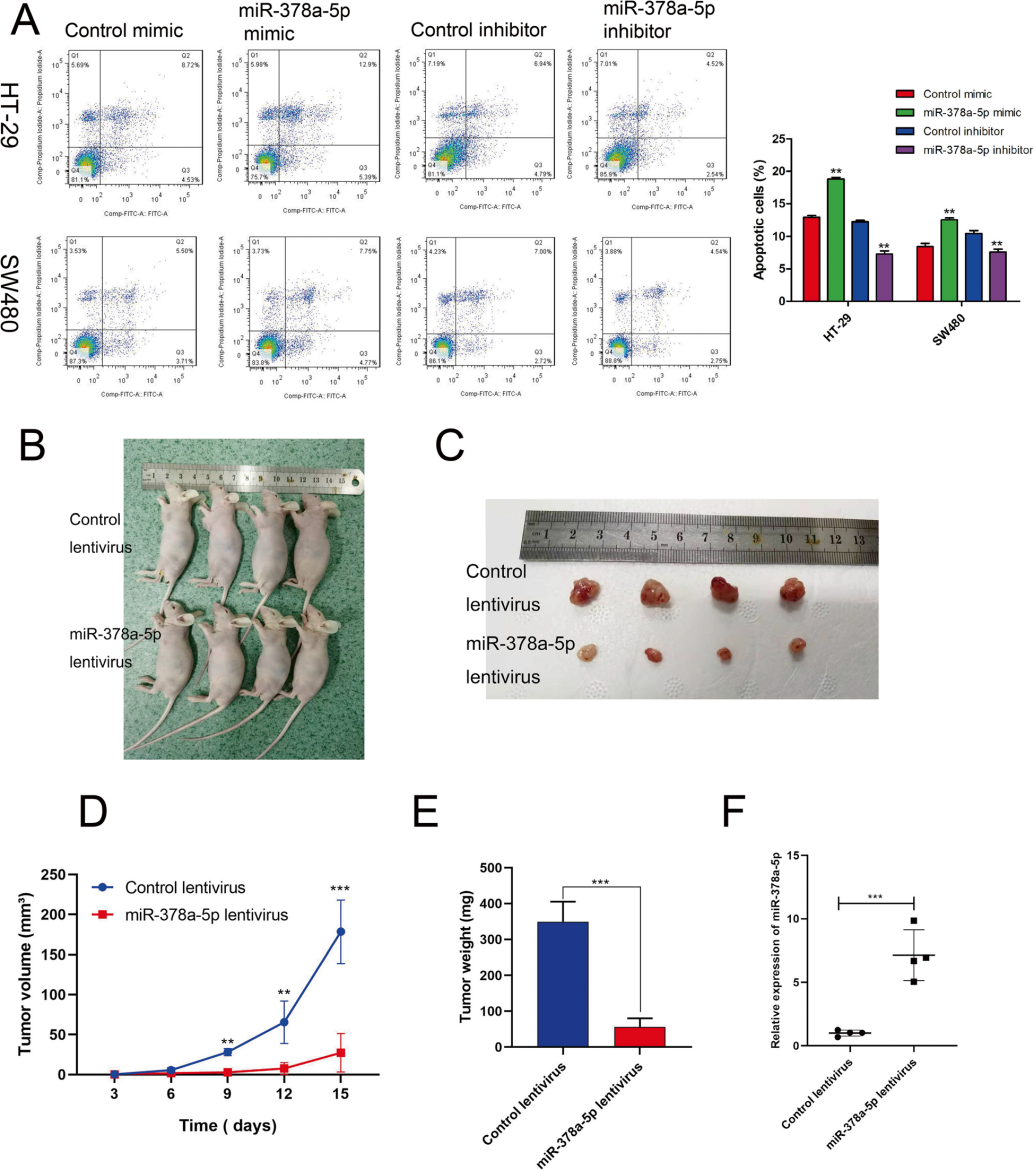
miR-378a-5p promotes colorectal cell apoptosis and inhibits tumor growth in vivo. **a** Flow cytometry apoptosis experiments were used to measure the apoptosis rate of HT-29 and SW480 cells. **b** Subcutaneous injection of HT-29 cells stably transfected with control lentivirus and miR-378a-5p lentivirus into the armpit of nude mice. **c** Nude mice were sacrificed 15 days later, and tumors were collected and recorded. **d** A vernier caliper was used to measure the tumor volume every 3 days (*n* = 4) to draw a tumor growth curve. **e** Xenograft tumor weight of miR-378a-5p-overexpressing (miR-378a-5p lentivirus) HT-29 cells and control (control lentivirus) HT-29 cells in a xenograft model (*n* = 4). **f** qRT-PCR was used to detect the relative expression of miR-378a-5p in xenograft tumor tissues, and the data are expressed as the mean ± standard deviation, *n* = 4 for each group. **p < 0.01; ***p < 0.001

The miR-378a-5p mimic and miR-378a-5p inhibitor were transfected into HT-29 and SW480 cells to overexpress and inhibit the expression of miR-378a-5p. Flow cytometry was used to assess the apoptosis rate, which revealed that the overexpression of miR-378a-5p promoted the apoptosis of colon cancer cells, while inhibition of the expression of miR-378a-5p inhibited the apoptosis of colon cancer cells (Fig. 3a). We subcutaneously inoculated nude mice with lentivirus or negative control stably infected HT-29 cells to clarify whether miR-378a-5p affects tumorigenesis in vivo (Fig. 3b). The results showed that miR-378a-5p significantly reduced tumor volume and weight (Fig. 3c, d) and that tumor growth was significantly slower (Fig. 3e). qRT-PCR analysis showed that the expression of miR-378a-5p in subcutaneous tumors formed by HT-29 cells overexpressing miR-378a-5p increased (Fig. 3f). Therefore, these results proved that overexpression of miR-378a-5p inhibited tumor growth in vivo.

### CDK1 is the miR-378a-5p target gene, with a high expression in CRC tissues

**Fig. 4:**
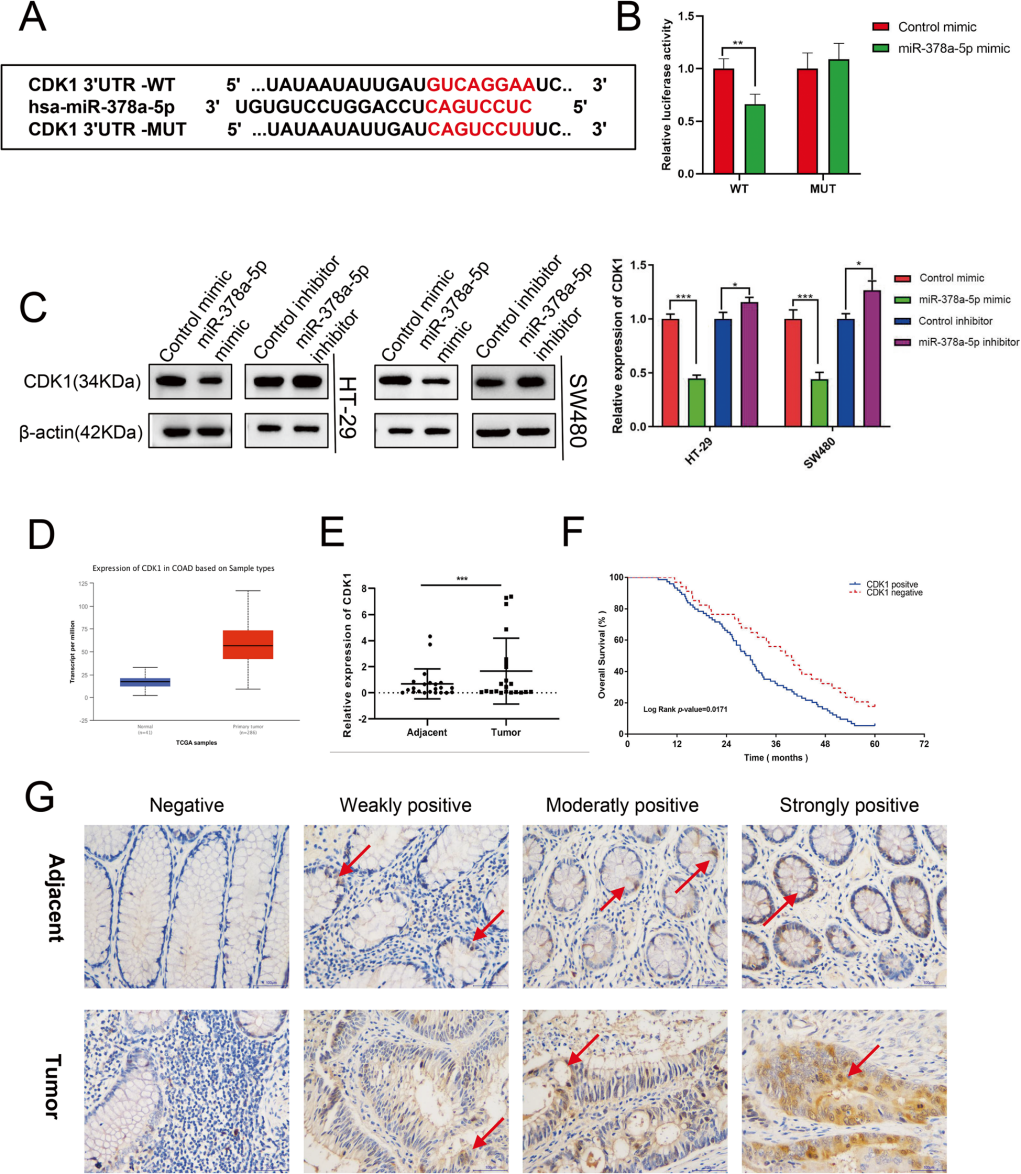
CDK1, a miR-378a-5p target gene, is overexpressed in CRC. **a** Target genes of miR-378a-5p were predicted using TargetScan and RNAhybrid. **b** A luciferase reporter assay was conducted to verify the binding association of miR-378a-5p with CDK1. **c** Western blotting for CDK1 protein levels following miR-378a-5p mimic/inhibitor transfection. **d** TCGA database analysis of CDK1 expression in CRC. **e** CDK1 expression in 22 paired CRC tissues and tumor-adjacent tissues measured by qPCR. **f** Kaplan-Meier survival analysis of the effect of CDK1 levels on the prognosis of CRC patients. **g** Negative, weakly positive, medium positive, and strongly positive results of CDK1 expression in CRC tissues determined by IHC. **p < 0.01; ***p < 0.001

To better investigate the mechanism underlying the impact of miR-378a-5p on inhibiting CRC cell proliferation and migration, TargetScan was used in combination with RNAhybrid for predicting miR-378a-5p target genes and revealing whether miR-378a-5p binds to CDK1 mRNA (Fig. 4a). To this end, we performed a luciferase reporter assay with the 293T cell line. According to our findings, overexpression of miR-378a-5p inhibited the fluorescence intensity in the unmutated 3′UTR of CDK1, while the miR-378a-5p gain-of-function did not change the fluorescence intensity in the 3′UTR of CDK1 in which the site of possible binding for miR-378a-5p was mutated, which suggested that miR-378a-5p bound to the 3′UTR of CDK1 mRNA (Fig. 4b). Furthermore, as shown by Western blotting, the miR-378a-5p level showed a negative correlation with CDK1 expression (Fig. 4c), further confirming that CDK1 was targeted by miR-378a-5p. Then, the CDK1 level was examined against TCGA database (Fig. 4d). Furthermore, the 22 CRC and adjacent noncarcinoma tissues were subjected to qPCR analysis (Fig. 4e), which revealed high CDK1 expression within CRC. As suggested by Kaplan-Meier survival analysis, CRC patients who had upregulated CDK1 expression had a markedly decreased OS rate compared with those with downregulated CDK1 expression (Fig. 4f). To elucidate the possible effect of CDK1 on CRC progression, IHC was performed to analyze the expression of CDK1 in paraffin-embedded tissues of 108 CRC patients from the Department of Pathology (Fig. 4g), and the associations of CDK1 level with patient clinicopathological features were examined, which indicated a high CDK1 expression within the CRC and had an aggravating effect on CRC. These results revealed that CDK1 was negative in 34 cases and positive in 74 cases, and the high expression level of CDK1 was related to tumors in the right colon (*p* = 0.029), lymph node metastasis (*p* = 0.020), and tumor-node-metastasis (TNM) stage (*p* = 0.031) (Table 1). Taken together, CDK1 was targeted by miR-378a-5p and was overexpressed in CRC.

**Table 1:**
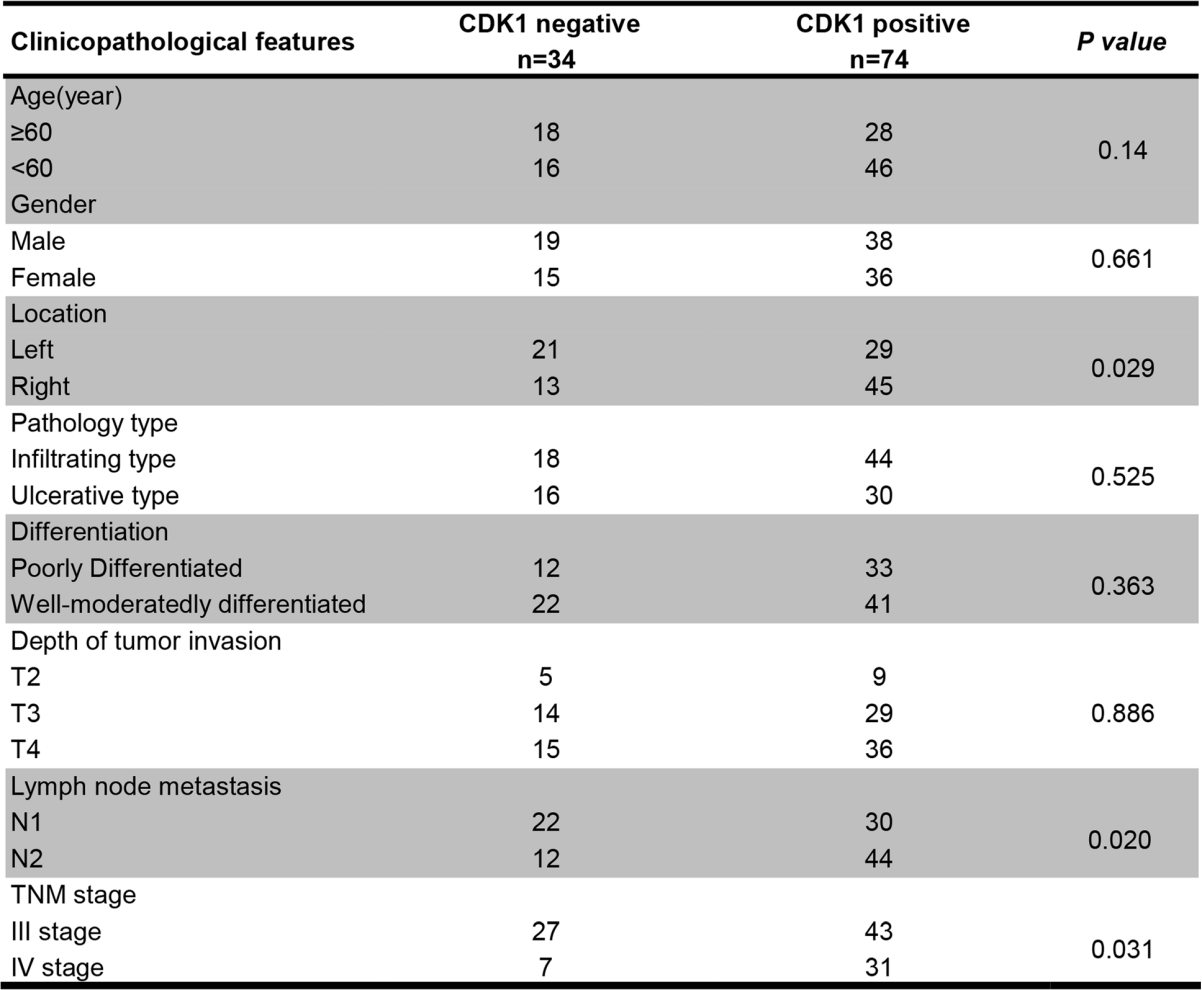
Clinicopathologic features of 108 patients with colorectal cancer

### CDK1 facilitates CRC cell proliferation and migration

**Fig. 5:**
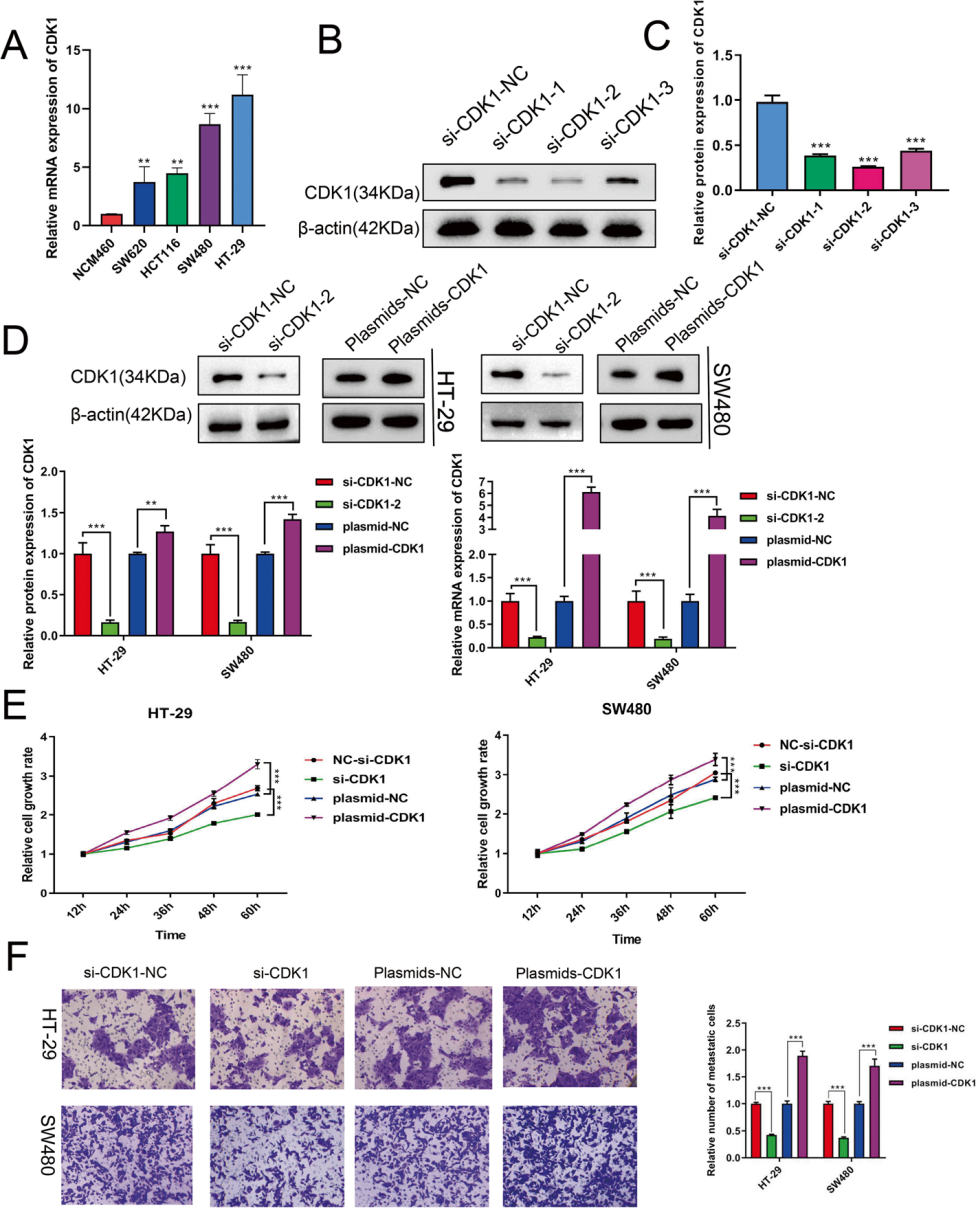
CDK1 stimulates the proliferation and migration of CRC cells. **a** CDK1 expression in NCM460, SW620, HCT116, HT-29, and SW480 cells determined by qPCR. **b**/**c** The silencing efficiency of the three siRNAs detected using Western blot analysis. **d** The silencing or overexpression efficiency of CDK1 was assessed by qPCR as well as Western blotting. **e** Relative CRC cell growth rates at 12 h, 24 h, 36 h, 48 h, and 60 h after knockdown or upregulation of CDK1 were measured using a CCK-8 assay. **f** Transwell assays were conducted to assess how CDK1 overexpression or knockdown affected the migration of CRC cells. **p **<** 0.01; ***p **<** 0.001

The expression of CDK1 in normal colon epithelial cells and CRC cells assessed by qPCR demonstrated that CDK1 was highly expressed in CRC cells (Fig. 5a). We used siRNA to knockdown CDK1 expression and constructed a CDK1 overexpression plasmid to overexpress CDK1 (Fig. 5b, c, d). Then, CRC cell growth rates at 12 h, 24 h, 36 h, 48 h, and 60 h after transfection were examined through the CCK8 assay to investigate how CDK1 overexpression or knockdown affected the CRC cells. The results showed that CDK1 knockdown suppressed CRC cell proliferation, whereas CDK1 overexpression accelerated CRC cell proliferation (Fig. 5e). Transwell assay results revealed that CDK1 loss-of-function reduced the number of migrating cells, while CDK1 upregulation stimulated the migration of CRC cells (Fig. 5f). In other words, CDK1 promoted CRC cell proliferation and migration.

### Upregulation of CDK1 restores the inhibitory effect of overexpressed miR-378a-5p on CRC cell proliferation

**Fig. 6:**
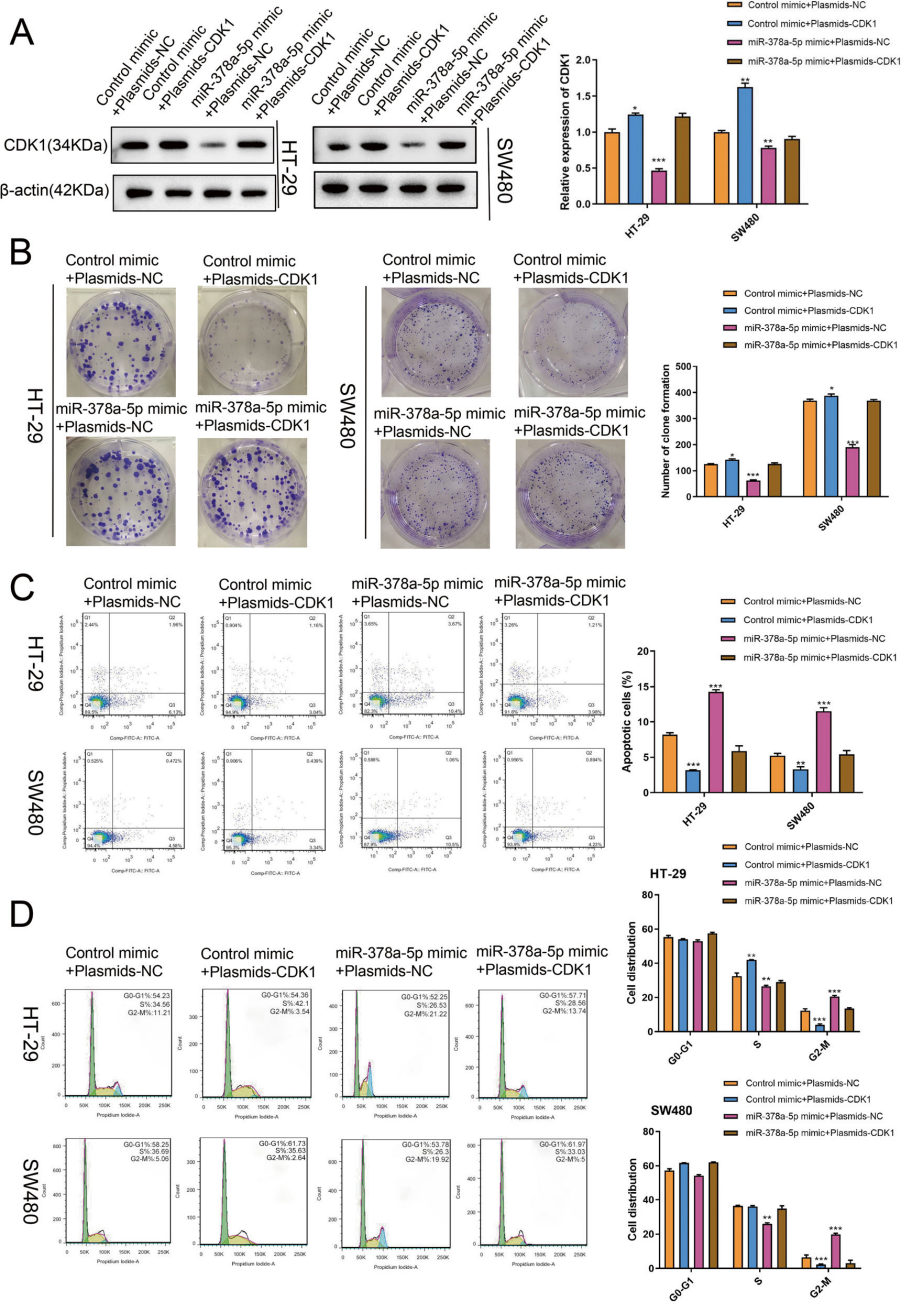
The gain-of-function of CDK1 reverses the inhibitory effect of miR-378a-5p overexpression on CRC cell proliferation. **a** Western blotting showed the CDK1 level within CRC cells. **b** A colony formation assay was carried out to assess how the diverse treatments affected the proliferation of CRC cells. **c**/**d** Flow cytometry was conducted to determine how diverse treatments affected CRC cell apoptosis and the cell cycle. **p < 0.01; ***p < 0.001

To further validate whether miR-378a-5p could target CDK1, we overexpressed miR-378a-5p and CDK1, alone or in combination, in CRC cells. As suggested by Western blotting analysis, the CDK1 level remarkably declined after miR-378a-5p was overexpressed, while CDK1 expression was similar to that in the control group after overexpression of miR-378a-5p and CDK1 together (Fig. 6a). As shown by the colony formation assay, colony number declined after overexpression of miR-378a-5p but increased after overexpression of CDK1; the colony number was similar to that of the control group when miR-378a-5p was coexpressed with CDK1 (Fig. 6b). It was illustrated by apoptosis assay that miR-378a-5p overexpression enhanced the CRC cell numbers at the G2-M phase and cell apoptosis, whereas CDK1 upregulation resulted in the opposite trend; the upregulation of miR-378a-5p and CDK1 together induced a trend similar to the control group (Fig. 6c, d). The above results supported that miR-378a-5p inhibited proliferation but promoted the apoptosis of CRC cells, while CDK1 reversed these effects; overexpressing CDK1 reversed the inhibition of miR-378a-5p overexpression on CRC cell proliferation, demonstrating the targeting relationship between the two.

## Discussion

CRC ranks 3rd in terms of its morbidity, second only to lung cancer and breast cancer. Recently, CRC morbidity has shown an increasing trend because of changes in lifestyle, increased obesity, and economic development [17]. Therefore, it is particularly important to elucidate the pathogenesis of CRC to improve the diagnosis and treatment of CRC, thereby reducing its mortality.

Our previous study found that LINC00365 promotes the development of CRC and that the expression of CDK1 increases with the upregulation of LINC00365, revealing that CDK1 may be related to the development of CRC [18]. In this experiment, it was found that CDK1 is closely related to the development of colorectal cancer. Overexpression of CDK1 promotes the proliferation, invasion, and migration of CRC cells, indicating that CDK1 has a promoting role in the development of CRC. Some researchers have found that CDK1 and cyclin B1 may be potential diagnostic biomarkers for rhabdomyosarcoma and hepatocellular carcinoma [19, 20]. As discovered by Yamamura et al., phosphorylated CDK1 (p-CDK1), p-CDK2, cyclin B1, and cyclin E1 levels increase within cholangiocarcinoma tissues; in addition, p-CDK1 and cyclin B1 nuclear levels positively correlate with the clinical stage and with lymph node metastasis, while the expression of p-CDK 1 is related to poor patient survival [21]. A recent study has also shown that CDK1 is closely related to autophagy [22]. Therefore, CDK1 may be an important biomarker and therapeutic target for CRC.

Noncoding RNAs are tiny RNA molecules that function in transcription and other specific processes, but they cannot encode proteins [23]. Among noncoding RNAs, miRNAs are overexpressed and mutated in a variety of malignant tumors, mainly by regulating the expression of mRNA and are considered to be important for the treatment of various diseases, especially cancer [23–27]. In this study, it was found that the expression of miR-378a-5p in CRC tissues decreased, and by overexpression or knockdown of miR-378a-5p, it was found that miR-378a-5p inhibited CRC cell proliferation and migration and promoted cell apoptosis. Through bioinformatics prediction and dual luciferase reporter gene detection, CDK1 was found to be the target gene of miR-378a-5p, revealing that miR-378a-5p may regulate the development of CRC cells by regulating the expression of CDK1. There are many unique noncoding RNA sequences in cells, among which lncRNAs are considered competitive endogenous RNAs (ceRNAs) that interact with microRNAs and participate in the regulation of target gene expression [28, 29]. Whether there is a lncRNA upstream of miR-378a-5p that participates in regulation is not yet known, and whether miR-378a-5p has the same regulatory effect in other tumors has not been studied. These questions will be explored in future studies.

In conclusion, we found that miR-378a-5p inhibited the proliferation of colorectal cancer cells, and CDK1 promoted the development of colorectal cancer. The findings in this work suggested that miR-378a-5p inhibits CRC cell proliferation by targeting CDK1, which can shed more light on CRC treatment.

## Data Availability

All authors ensure that our data does not contain any of the following: (a) Infringes or makes unauthorized use of the Intellectual Property Rights or any other right of any person; (b) Is defamatory, derogatory, discriminatory, or violates any rights of privacy; (c) Breaches any applicable law or regulation; (d) Contains a virus, malware, or other potentially harmful component, information, or instructions; (e) Is indecent, obscene, or offensive. Please confirm agreement with this statement.
